# Ectothermy and cardiac shunts profoundly slow the equilibration of inhaled anaesthetics in a multi-compartment model

**DOI:** 10.1038/s41598-020-74014-y

**Published:** 2020-10-13

**Authors:** Catherine J. A. Williams, Christian Lind Malte, Hans Malte, Mads F. Bertelsen, Tobias Wang

**Affiliations:** 1 Section of Zoophysiology, Department of Biology, Aarhus University, 8000 Aarhus C, Denmark; 2 Center for Zoo and Wild Animal Health, Copenhagen Zoo, Roskildevej 38, 2000 Frederiksberg, Denmark; 3 Ontario Veterinary College, University of Guelph, 50 Stone Road E, Guelph, ON N1G 2W1 Canada; 4 Aarhus Institute of Advanced Sciences, Aarhus University, 8000 Aarhus C, Denmark

**Keywords:** Physiology, Cardiovascular biology, Respiration, Computational models

## Abstract

The use of inhalational anaesthesia is ubiquitous in terrestrial vertebrates. Given the dependence of these agents on delivery by the cardiorespiratory system, we developed a new computational model predicting equilibration of inhaled anaesthetics in mammalian and ectotherm conditions including the ability of reptiles to maintain vascular shunts. A multi-compartment model was constructed from simultaneously-solved equations, verified by comparison to the literature for endo and ectotherm physiology. The time to 90% equilibration of anaesthetic in arterial blood (t_90_) is predicted and used to compare anaesthetics and physiologies. The five to tenfold lower cardiac output and minute ventilation of ectothermic vertebrates is predicted to slow equilibration times by five to ten times leading to 90% equilibration in ectotherm arterial blood of over 200 min, compounded by reduction in body temperature, and the extent of right-to-left vascular shunts. The impact of these findings is also influenced by the solubility coefficient of the anaesthetic, such that at net right-to-left shunt fractions of over 0.8, sevoflurane loses the advantage of faster equilibration, in comparison with isoflurane. We explore clinical strategies to regulate anaesthetic uptake in ectotherms by managing convectional flow especially by supportive ventilation and reduction of the right-to-left shunt.

## Introduction

Given its ubiquitous efficacy, inhalation anaesthesia is broadly used in medicine, veterinary medicine and research^1–3^, and the potency of these anaesthetics is typically evaluated from the minimum anaesthetic concentration (MAC) required to eliminate purposeful movement in response to nociceptive stimuli in 50% of a population^4^. It has, however, proved frustratingly difficult to quantify MAC in reptiles^5,6^. Using a mathematical description of anaesthesia uptake and elimination, we demonstrate that the difficulties in determining MAC in these ectothermic vertebrates, at least in part, are due to their low minute ventilation, low cardiac output and cardiac shunts.

The anaesthetic effect of volatile agents depends on their ability to reach the central nervous system (CNS) via inhalation and transport by arterial blood. The high minute ventilation ($\dot{V}_{L}$) of mammals enables fast onset and elimination of volatile anaesthetics, such that anaesthetic depth is easily adjustable, whereas the low minute ventilation of ectotherms is likely to slow the rate of equilibration^7,8^. The influence of cardiac output ($\dot{Q}_{tot}$) is more complex as high cardiac output quickly depletes the lungs of inhalation anaesthetic and hence may delay delivery to the CNS and anaesthetic induction, whilst high cardiac output obviously also hastens overall delivery and hence promotes eventual equilibration^1,9^. The interplay between variations in minute ventilation and cardiac output on induction and equilibration of inhalation anaesthetics is amenable to mathematical modelling^10,11^ and can provide relevant information for clinicians and experimental scientists.

In addition to the low rates of ventilation and cardiac output of reptiles, their undivided ventricle (or extra-ventricular shunt in crocodilians) also provides for sizable cardiovascular shunts where particularly the presence of right-to-left (R–L) shunts (systemic venous blood that bypasses the pulmonary circulation) may hinder the uptake of inhaled anaesthetics to the blood^5,12,13^. Indeed, Greunz et al. (2018) recently showed that pharmacological reduction of a net cardiac R–L shunt led to determination of a lower MAC, reflective of the closer equivalence of inhalation anaesthetics in the arterial blood and end-tidal air. These observations are consistent with model calculations in humans that predict slower rates of uptake and elimination in the presence of pulmonary ventilation-perfusion (V/Q) mismatch and, in the case of certain congenital heart defects, levels of cardiac R–L shunt^11,14^. The influence of R–L shunt depends on the solubility of the inhalation anaesthetic in blood, where gases with low solubility are disproportionally more affected by cardiac shunts, although low solubility per se provides for faster equilibration in the CNS^11,14^. Reptiles can exhibit very large R–L shunts^15–18^, but the influence of shunts on inhalation anaesthesia has never been quantified using relevant values for cardiac output ($\dot{Q}_{tot}$), and minute ventilation ($\dot{V}_{L}$).

In the present study, we constructed a model to predict partial pressures of anaesthetic gases in multiple compartments of the cardiorespiratory system including lung, arterial blood, venous blood and tissue to evaluate the effects of realistic and physiologically relevant values for anaesthetic solubility, cardiac output, minute ventilation and R–L shunts on the rate of equilibration. We use the partial pressure in the arterial blood compartment to indicate the partial pressure in the central nervous system, as it is such a highly perfused, low mass organ^19^. This theoretical approach allows us to address (1) the influence of much lower $\dot{V}_{L}$ and $\dot{Q}_{tot}$ in ectotherms compared with those in mammals, and (2) how cardiac shunts—the normal condition in the reptilian cardiovascular system—influence uptake, equilibration and elimination of inhaled anaesthetics. Hence, we systematically describe the effects of $\dot{V}_{L}$, $\dot{Q}_{tot}$ and shunt on an anaesthetic profile using realistic physiological parameters and verified by comparison to clinical data. Modelling anaesthetic uptake and elimination allows exploration and explanation of some previously anomalous findings in reptile anaesthesia: the recovery from and deepening of anaesthesia hypothesised to be associated with peri-surgical shunt changes, the difficulty in measuring MAC in reptiles, and the apparent reduction in MAC over the time of an anaesthetic^6,20^. This is especially relevant for exotic veterinary medicine where sevoflurane, with one of the lowest solubilities of the inhaled anaesthetics, and hence most sensitive to cardiac shunts, has recently become a favored choice for anaesthesia.

## Results

**Figure 1:**
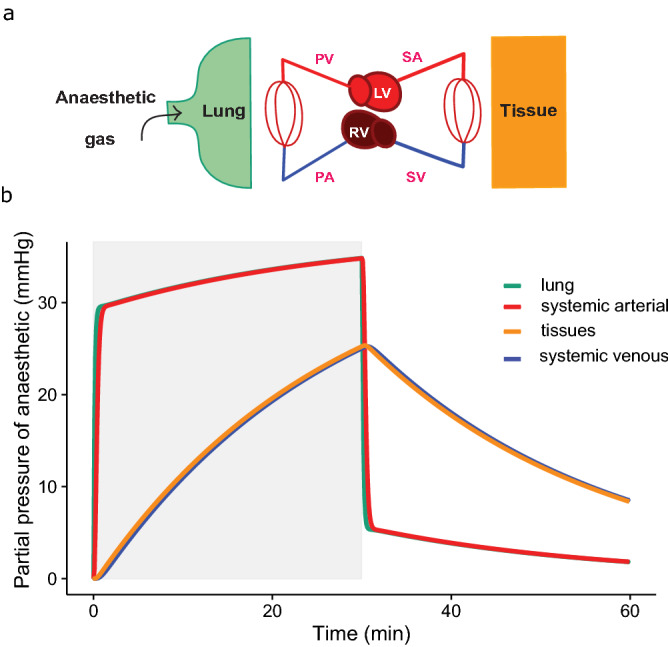
(**a**) Schematic of movement of anaesthetic gas from the lungs via the lung capillary bed (CL) into the circulation, tissue capillary bed (CT) and tissue. (**b**) Graph shows predicted uptake and washout over time of an exemplar gas (isoflurane) via partial pressures in the lung (L—turquoise), systemic arterial (SA-bright red), tissues (T-yellow) and systemic venous (SV—blue) compartments. The period of anaesthetic exposure is highlighted in light grey. Please see Table 1 for input cardiac and ventilatory variables. At the time resolution shown, the proximity of the values for lung and arterial blood and later for tissues and venous blood, lead to the traces for these predictions overlapping each other on the graph.

**Figure 2:**
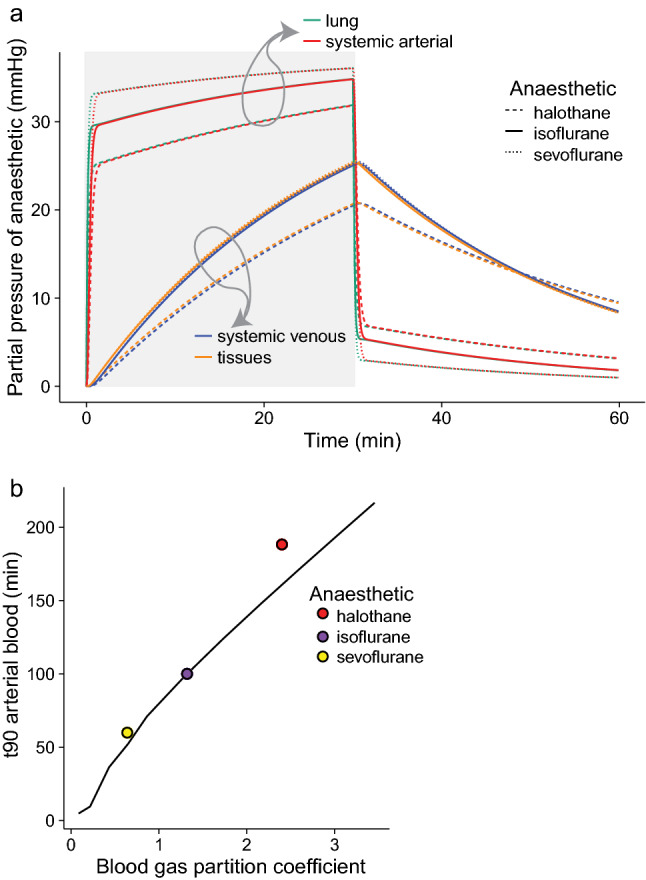
(**a**) Partial pressures of anaesthetic in the lung (turquoise), systematic arterial blood (bright red), systemic venous blood (blue) and tissues (yellow) in a 1 kg rabbit (see Table 1 for input variables) over a 30 min anaesthesia with 5% initial vaporizer setting (i.e., 37.5 mmHg) using halothane (dashed lines), isoflurane (solid line), and sevoflurane (dotted line). The period of anaesthetic exposure is highlighted in light grey. The time to 90% equilibrations in arterial blood is 7.3, 0.8, and 0.7 min respectively. Washout out over 30 min is also shown. Please see Table 1 for input cardiac and ventilatory variables. Where values are highly proximate (e.g., for partial pressures in lung and arterial blood) the lines on the figure may lie over one another. (**b**) Predicted time to 90% equilibration (t_90_) of anaesthetics in arterial blood plotted against their blood gas partition coefficient including data for halothane (red), isoflurane (purple), and sevoflurane (yellow), in an animal with longer equilibration times. (**—**) represents t_90_ with blood gas partition coefficient varying with a constant ratio of blood-gas to tissue-gas partition coefficients, based on isoflurane. Data points for halothane, isoflurane and sevoflurane use their measured blood gas, and tissue gas partition coefficients ^21,23,28,29,51^.

The model predicts the temporal development of the partial pressure of inhaled anaesthetics in all calculated compartments; Fig. 1a is a schematic of the circulation and Fig. 1b presents predicted partial pressures in lung, systemic arterial blood, tissue and systemic venous blood. The model predicts rapid equilibration in the lung and arterial blood, and slower equilibration with tissue and venous blood (Fig. 1b). Within the lung and arterial blood there is a fast first phase of uptake over the first minute followed by a slower phase until equilibration is reached. The washout phase is also shown for the first 30 min after the removal of anaesthetic from the inhaled gas. Figure 2a exemplifies this for a modelled mammal (rabbit) anaesthetized for 30 min with sevoflurane, isoflurane or halothane at 5% in the inhaled gas. The end point relevant for anaesthesia must lie between that of arterial blood and the mixed tissue compartment. The central nervous system is a highly perfused, low mass organ both in mammals and reptiles^19^, therefore the equilibration time in arterial blood is considered as the variable best representing the onset of effective anaesthesia in the model. The time to 90% equilibration in arterial blood (t_90_) is 7.3, 0.8, and 0.7 min, respectively, for halothane, isoflurane and sevoflurane, with the differences being dictated by their progressively smaller blood gas partition coefficients. These coefficients describe the solubility of an anaesthetic, the blood gas partition coefficient being the ratio of the solubility of the anaesthetic in the blood to that in the gas^21^. In the equations for the model we express them as capacitance coefficients, expressed in (mLGasSTPD/mL/mmHg), see Methods section.

The model predictions can then be compared with published clinical data: with input of 53.5 mmHg sevoflurane, equivalent to a chamber or mask induction with 7% sevoflurane, the surgical MAC in arterial blood (3.7% or 28 mmHg for the rabbit) is reached in under 30 s. Figure 2b, compares the anaesthetic agents in an animal with longer equilibration times (here a terrapin), where time to 90% equilibration in arterial blood is plotted against blood gas partition coefficient. Here, with a lower $\dot{Q}_{tot}$ and $\dot{V}_{L}$ than that of the rabbit, equilibration times are longer and the difference between t_90_s for the anaesthetics are more marked, increasing time to equilibration with increasing blood gas partition coefficient. Thus, the model can also be used to predict anaesthetic equilibration in ectotherms, e.g., for a varanid lizard induced at 8% sevoflurane, the model predicts MAC (2.4%) in arterial blood to be reached in 4 min at 37 °C, which compares well with clinical data of 10.4 min to induction at 27 °C, given a Q_10_ of 2–3^22^. In Fig. 2b, where three specific anaesthetic agents were compared overlying the predicted relationship between blood gas partition coefficient and time to arterial equilibration,—the data was modelled using the capacity coefficients (β_b_ and β_T-_) for isoflurane and then varying the coefficients while keeping the ratio of β_b_ to β_T_ constant. Thus, the exact predictions for halothane and sevoflurane fall slightly off the modelled curve, as their ratio βb to βT varies from that of isoflurane.

**Figure 3:**
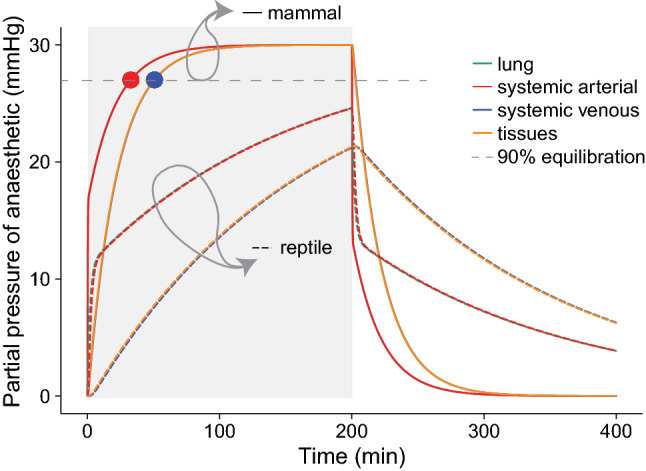
Anaesthetic equilibration in a representative mammal and reptile (Table 1 for input variables) anaesthetized with isoflurane with an inhaled partial pressure of 30 mmHg. The period of anaesthetic exposure is highlighted in light grey. In the mammalian example the t_90_ (time to 90% equilibration) is shown with overlap for the lung (turquoise,  ) and systemic artery (bright red,  ), and systemic venous (blue,  ) and tissue (yellow,  ) pools. In the reptilian example, none of the compartments equilibrate within a 200 min simulated anaesthetic. No vascular shunting was included in this model run (see Fig. 5 for the effect of shunt).

**Figure 4:**
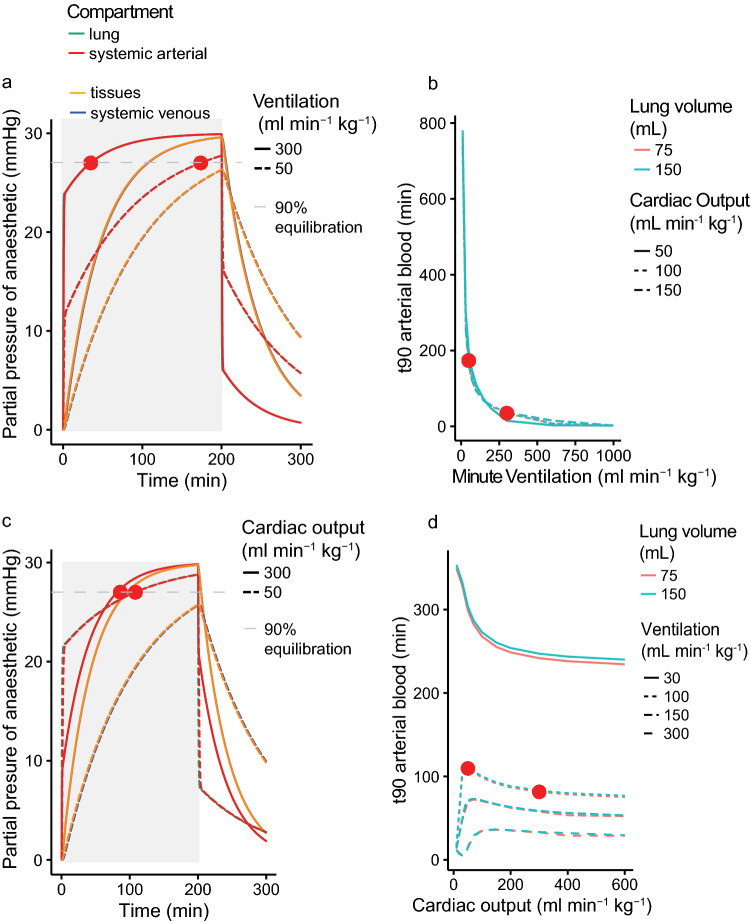
(**a**) The effect of minute ventilation on time to 90% equilibration (t_90_) of isoflurane. The period of anaesthetic exposure is highlighted in light grey. Two predicted equilibrations are shown for typical reptilian (dashed line) and mammalian (solid line) minute ventilations (50 and 300 ml min^−1^ kg^−1^ respectively) with t_90_ in arterial blood marked with  . Predicted values are shown for systemic arterial blood (red) and tissues (yellow) which at this magnification overlap with those for lung (turquoise) and systemic venous blood (blue) respectively. (**b**) The effect of varying ventilation over a wide range of values (x axis, 10–1000 ml kg^−1^ min^−1^) on time to 90% equilibration in arterial blood (y axis). Predicted values are shown for three different cardiac outputs (50, 100 and 150 ml min^−1^ kg^−1^, solid, dotted and dashed lines respectively) and two different lung volumes (75 and 150 mL, red and blue respectively). The t_90_ for the cases exemplified in (**a**) are again shown as  . (**c**) The effect of varying cardiac output on time to 90% equilibration (t_90_). The period of anaesthetic exposure is highlighted in light grey. Two predicted equilibrations are shown for typical reptilian (dashed line) and mammalian (solid line) cardiac outputs (50 and 300 ml min^−1^ kg^−1^ respectively) with t_90_ in arterial blood marked with  . Predicted values are shown for systemic arterial blood (red) and tissues (yellow) which at this magnification overlap with those for lung (turquoise) and systemic venous blood (blue) respectively. t_90_ in arterial blood for each case is marked with  . (**d**) The effect of varying cardiac output over a wide range of values (x axis, 10–600 ml kg^−1^ min^−1^) on time to 90% equilibration in arterial blood (y axis). Predicted values are shown for four different minute ventilations (30, 100, 150 and 300 ml min^−1^ kg^−1^, solid, dotted and various dashed lines respectively) and two different lung volumes (75 and 150 mL, red and blue respectively). The t_90_ for the cases exemplified in (**c**) are again shown as  . No vascular shunting was included in this model run (see Fig. 5 for the effect of shunt).

Figure 3 compares the partial pressure for isoflurane between a representative mammal and reptile, where the tissue mass, blood volume and tissue and blood solubilities are the same, but where lung volume (L), cardiac output ($\dot{Q}_{tot}$) and minute ventilation ($\dot{V}_{L}$) are set at representative levels (Table 1). Taking t_90_ as the outcome of the model, reptilian physiology profoundly slows the equilibration, leading to ten times longer equilibration times (mammal t_90_ = 31 min , reptile t_90_ = 294 min , thus not within the 200 min anaesthetic shown in Fig. 2), shifting both phases of the equilibration curves down and to the right. In this example, $\dot{V}_{L}$, $\dot{Q}_{tot}$ and lung volume represented reptilian and mammalian values. Hence, we then explored the individual influence of physiology and clinical factors on the uptake of anaesthetic; Fig. 4a–d show the effects of $\dot{V}_{L}$, $\dot{Q}_{tot}$, and L on t_90_ in arterial blood.

**Table 1:** (a) Initial model parameters are shown for the included figures, (b) Model parameters are shown for the included figures.

Figure	$\dot{Q}_{{tot}}$	$\dot{V}_{L}$	L	$\beta _{b}$	$\beta _{T}$	R–L shunt	L–R shunt	Inspired partial pressure of anaesthetic	
	mL	mL kg^−1^ min^−1^	mL	mLSTPD mL^−1^ mmHg^−1^	mLSTPD mL^−1^ mmHg^−1^			mmHg	
1a. ‘rabbit’	175	500	75	0.01528	0.00461	0	0	35	
2a. ‘rabbit’	175	500	75	0.00074–0.00278	0.0025–0.0099	0	0	35	
2b.	100	100	150	0.0001–0.004	0.00302–0.012064	0	0	30	
3a ‘mammal’	300	300	75	0.001528	0.00461	0	0	30	
‘reptile’	60	30	150	0.001528	0.00461	0	0	30	
4a.	50, 300	100	150	0.001528	0.00461	0	0	30	
4b.	30–150	10–1000	75–150	0.001528	0.00461	0	0	30	
4c.	100	50, 300	150	0.001528	0.00461	0	0	30	
4d.	10–600	30–300	75–150	0.001528	0.00461	0	0	30	
5a.	100	100	150	0.00074–0.00278	0.0025–0.0099	0–0.9	0–0.9	30	

	T	CT	CL	PV	PA	SA	SV	RV	LV
	mL	mL	mL	mL	mL	mL	mL	mL	mL
1-5. inclusive	600	0.15	0.15	12	7.5	15	37.5	0.75	0.75
Terrapin	400	0.1	0.1	8	5	10	25	0.5	0.5

$\dot{V}_{L}$ had a profound effect on the rate of equilibration. Figure 4b shows the relationship between $\dot{V}_{L}$ and t_90_ in arterial blood, where increased $\dot{V}_{L}$ decreased the time to equilibration over the physiological and clinical range, at a range of $\dot{Q}_{tot}$ and lung volumes.

Clinically, where total equilibration is not required for anaesthetic effects to be apparent, Fig. 4c (plotted again for isoflurane) shows that up until ~ 85% equilibration, lower cardiac output is associated with a faster uptake of anaesthetic, as expected from the human and veterinary literature^9^ i.e., there is a faster rise in arterial partial pressure in the 50 ml min^−1^ kg^−1^ $\dot{Q}_{tot}$ prediction than that for 300 ml min^−1^ kg^−1^. Taking 90% equilibration as our outcome measure (t_90_) the relationship between $\dot{Q}_{tot}$ and t_90_ was more complex (Fig. 4c); at $\dot{Q}_{tot}$ of over 100 ml min^−1^ kg^−1^ there was a relatively small influence on arterial time to equilibration, with increased $\dot{Q}_{tot}$ leading to faster arterial equilibration. However, at lower $\dot{Q}_{tot}$, the direction of the relationship was dependent on $\dot{V}_{L}$. At low (30 ml min^−1^ kg^−1^) $\dot{V}_{L}$, increasing $\dot{Q}_{tot}$ hastened arterial equilibration. Conversely, as clinically predicted, at high $\dot{V}_{L}$, a low $\dot{Q}_{tot}$ lead to faster arterial equilibration, as the tissue pool was increasingly isolated and anaesthetic did not move into it, hence the arterial pool equilibrated faster, and the low $\dot{Q}_{tot}$ allowed a higher partial pressure to build in the lungs^1^.

**Figure 5:**
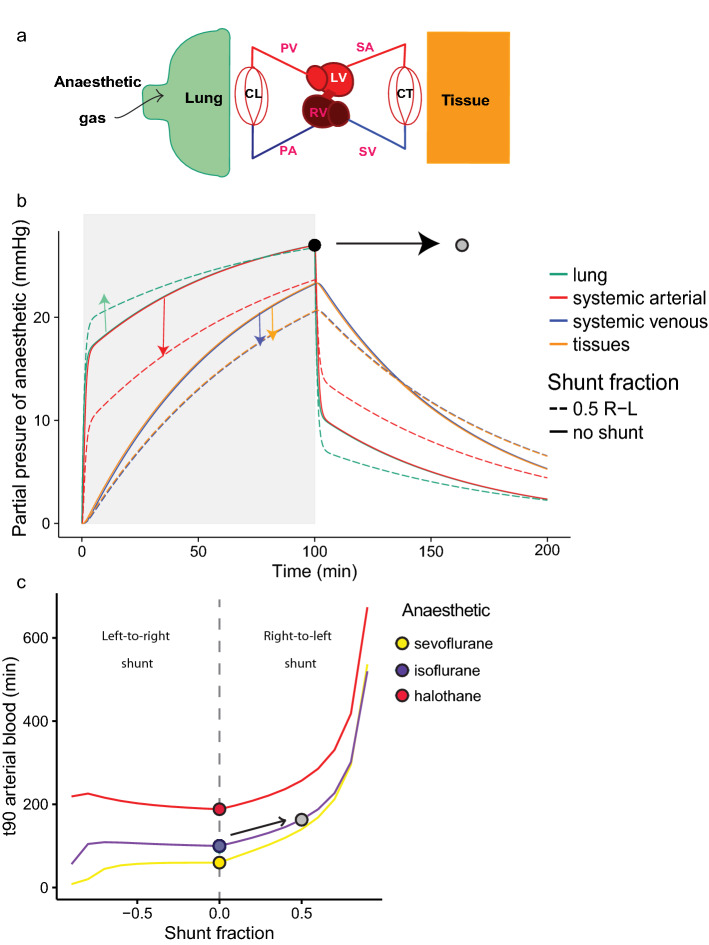
(**a**) Schematic diagram of a circulation with intraventricular shunting, as is typical for non-crocodilian reptiles. (**b**) The effect of a 50% shunt from the pulmonary to systemic circulation (0.5 right-to-left shunt), is shown in lung (turquoise), systemic arterial blood (red), tissue (yellow) and systemic venous blood (blue). The period of anaesthetic exposure is highlighted in light grey. Although equilibration in the lung is faster in the shunting condition (green arrow), the partial pressure of anaesthetic in the arterial blood (red arrow) moves away from that in the lung, thus slowing equilibration, such that 90% equilibration is not attained in the arterial blood after 100 min of anaesthesia with a shunt ( ), while it is in the non-shunting condition ( ). (**c**) t_90_ in arterial blood (y-axis) at varying degrees of intraventricular shunt (x-axis). Predictions are shown for anaesthetics with differing blood gas capacitance coefficients (*b*_*b*_), and exemplified for halothane (red), isoflurane (purple) and sevoflurane (yellow). The case in (**b**) is presented as ( ).

Lung volume had little influence on anaesthetic gas equilibrium per se (Fig. 4b,d), but the combination of low $\dot{V}_{L}$ and their large lungs contribute to the slower equilibration in reptiles (Fig. 3). The model predicts major effects of shunts (as presented in the schematic in Fig. 5a) because the arterial anaesthetic partial pressure (which bathes the central nervous system) becomes lower than that in the lung (red and turquoise arrows in Fig. 5b). Thus, when 50% of blood bypasses the pulmonary circulation (expressed in the model as a right-to-left shunt of 0.5), t_90_ in arterial blood of isoflurane is lengthened by more than 50%, and the time required for tissue equilibration is prolonged. Figure 5c shows the influence of shunt on time to equilibration and demonstrates the predicted dependence of this effect on the solubility of the gas anaesthetic, as indicated for isoflurane, halothane and sevoflurane and represented by the plotted circles for the same shunt as modelled in Fig. 5b.

### Further model predictions

Three modifications to the model were considered, but given their low influence on the predictions, their effects are not reported.

1. Lower haematocrit in ectotherms tends to slightly reduce the blood gas capacitance coefficients and therefore increase equilibration times^23^; a reduction of hematocrit from typical human values to 20, caused a < 3% increase in equilibration time.
2. The model also allows for bidirectional shunts, i.e., the physiologically realistic scenario in non-crocodilian reptiles where left-to-right and right-to-left shunt occur concurrently^24–26^. In the case of anaesthetic uptake and elimination, while the mixing in the heart engendered by bidirectional shunting minimally slows equilibration, the t_90_ is mostly tethered to the degree of right-to-left shunt, as would be expected given inhaled anaesthesia’s mode of entry via the lungs and thus reliance on pulmonary blood flow.
3. As we use arterial blood equilibration as the outcome variable of interest in this paper, the equilibration times presented represent conservative times to equilibration at the CNS. Model predictions therefore if anything underrepresent the probable effects of ectothermy and shunt on anaesthetic uptake. This approach is justified for uptake as the CNS is a low mass, highly perfused organ in both mammals and reptiles^19,27^. Previous papers examining human inhaled anaesthetic pharmacokinetics have modelled an additional ‘vessel-rich’ compartment^10,11^, this was not performed here to aid the comprehension of the model, as its inclusion requires assumptions to be made of additional varying factors of tissue perfusion and organ masses, which can hinder the transparency of the model’s interpretation regarding the effect of ventilation, cardiac output and cardiac shunts^10^. Factors affecting cerebral blood flow, such as the perfusion reduction secondary to hyperventilation, will also affect equilibration times in the CNS, as well as the initial rise in partial pressures of inhaled anaesthetics. In terms of elimination, after a long anaesthetic, a certain concentration of anaesthetic would reside especially in lipid dense tissues. It would therefore also be interesting to consider the influence of the low perfusion rate of adipose tissue: subcutaneous fat in mammals, and intrabdominal fat bodies in reptiles, on the elimination of anaesthetic from these lipid sinks (Tanner 1985).
4. For simplicity, tissues are represented by one compartment which here was constructed from a weighted average of the tissue solubilities for humans and body composition of rabbit. The partition coefficients for tissues are highly dependent on their fat content, given the high solubility of volatile anaesthetics in fat, producing significant differences even within one species, humans, depending on age and diet^21,28^. Tissue equilibration times, therefore, vary with body condition, tissue fat content, as well as species-specific blood gas partition coefficients^29^. Temperature effects on partition coefficients are also present, and may contribute to the predictions presented here giving a conservative estimate of the effects of ectothermy on equilibration, as most ectotherms’ preferred temperature zones are below the core body temperature of most mammals, leading to marginally higher partition coefficients and thus longer equilibration times at lower temperatures. The decision was made here to show data for partition coefficients at 37° to ease the comparison between reptilian and mammalian conditions.

## Discussion

In line with our hypotheses, our model clearly predicts that low $\dot{V}_{L}$ greatly prolongs the time required for inhalation anaesthesia to reach equilibration in the body compartments as well as slowing the initial rate of uptake. Our model also predicts that right-to-left shunts significantly slow the equilibration of inhalation anaesthetics. Reptiles’ cardio-respiratory physiology, therefore, lengthens the time until the CNS experiences a partial pressure of anaesthetic provided in the lung and hence renders reptiles less immediately sensitive to changes in inhaled anaesthesia than mammals. These findings are relevant for the formulation and improvement of clinical anaesthesia protocols.

### The influence of convective blood flow: cardiac output

The combination of low $\dot{Q}_{tot}$ and $\dot{V}_{L}$ in ectothermic vertebrates profoundly slow arterial and tissue equilibration of inhaled anaesthetics in comparison with a mammal (Fig. 3). This is despite low $\dot{Q}_{tot}$ acting to hasten onset of anaesthesia, while lengthening eventual equilibration. This difference between the effects of high cardiac output on equilibration and initial onset of anaesthesia is consistent with the existing theoretical framework, where, high cardiac output is also predicted to hasten final CNS equilibration over the longer term^9^. However, in the early phase of anaesthesia, high cardiac output is predicted to prolong induction. This is because high convective flow away quickly depletes the lung of anaesthetic agent, effectively delivers it for sequestration in the tissue and hence delays the rate at which the partial pressure of anaesthetic builds up around the CNS. Also, a greater cardiac output with increased flow to usually low perfusion vascular beds will increase the exposure of the anaesthetic to tissue with a high anaesthetic gas solubility e.g., fat bodies, thus increasing the ‘sink’ for anaesthesia in the tissues. Since the CNS is highly perfused, we used the predicted arterial partial pressure as a proxy for partial pressure in the CNS (Fig. 4). We predict that low cardiac output has a fast first phase of rise in arterial partial pressure, and an earlier onset of the second slow phase, due to movement of anaesthetic into the tissues.

In summary, using typical reptilian values of a fivefold lower $\dot{Q}_{tot}$ and tenfold lower $\dot{V}_{L}$ (Table 1), we predict that equilibration will take over five times longer than in a mammal (Fig. 3). Thus, the long time required for equilibration in reptilian arterial blood rarely falls within the time frame of a normal clinical anaesthesia.

### Artificial ventilation can hasten equilibration

Reptiles often lose respiratory drive at surgical depths of anaesthesia and artificial ventilation is typically required, but needs to be matched to the low reptilian metabolism to maintain normal PaCO_2_^30,31^. However, given the steep dependence of t_90_ on $\dot{V}_{L}$ (Fig. 4b), it is clear that low ventilation, even at normal reptilian values, profoundly slows equilibration. This is especially true for anaesthetics with high solubility (i.e. high blood capacitance coefficients or blood gas partition coefficients) because they are less limited by blood convective transport. It may therefore be advisable to mildly over-ventilate reptilian lungs to hasten both onset and elimination of anaesthesia^30^. In research, although difficult in clinical practice, this can be combined with supplementing the inspired gases with CO_2_ to avoid respiratory alkalosis^30,32^_._

### The effect of temperature on anaesthetic equilibration

Within a given individual or species, equilibration times will be longer when $\dot{V}_{L}$ decreases 2–3 times when temperature is reduced by 10 °C (the “Q_10_ effect”)^33–36^. This underlines the importance of maintaining body temperature during anaesthesia both in ectotherms in general, but also in endotherms where temperature regulation under anaesthesia is blunted. The physiological consequences of hypothermia are amplified by temperature effects on the solubility of anaesthetics in tissue and blood, as documented in humans^23,28^. For simplicity, our model predictions are based on blood and tissue capacity coefficients at 37 °C to highlight the individual effects of $\dot{Q}_{tot}$ and $\dot{V}_{L}$. The effect of reducing temperature to 25 °C on blood/gas and tissue/gas partition coefficients^28^ prolongs arterial blood equilibration times by 12–13% in both mammals and reptiles, independent of the effects on convective flow.

### The effect of intraventricular shunting

Given their cardiac anatomy, vascular shunts are normal for reptiles^15,17,37^, and our model predicts that a R–L shunt significantly slows the uptake of gas into the blood and tissue compartments. This is because R–L shunts are associated with lower pulmonary perfusion and hence lower convective transport of the anaesthetic from the lungs. In the absence of shunts, alveolar arterial blood partial pressures of anaesthetic are almost identical (Fig. 5b solid turquoise and red lines). In the presence of R–L shunt, however, the partial pressure of the anaesthetic is lower than within the lungs (the divergence of red and turquoise dashed lines, Fig. 5b), which prolongs the time to equilibration. The influence of right-to-left shunts has been previously modelled^11,14^ and clinically reported in dogs and humans^38–40^. However, the normal range of right-to-left shunts in reptiles are considerably larger than those due to mammal cardiac malformations. Furthermore, given the long equilibration times in reptiles, relative prolongation due to R–L shunts have larger impact.

Anaesthetics with lower blood gas partition coefficients, such as sevoflurane, are more affected by right-to-left shunts, as their uptake is more sensitive to the pulmonary blood flow, than to $\dot{V}_{L}$^11,14^. This effect was predicted by Eger and Severinghaus (1964) but the ventilation perfusion mismatch modelled therein and shunts modelled by Tanner are smaller than those common in reptiles (25 and 50% in comparison with ~ 80% shunt under isoflurane in red footed tortoises^6^). Our model therefore predicts a reversal in the equilibration of isoflurane and sevoflurane, at high shunts (R–L of > 0.8, Fig. 5c) that was not predicted for cyclopropane and ether in a mammalian system.

Over all, the effects of shunts, while significant at high right-to-left shunt fraction, are in most situations less influential than a reduction in convective flow on the equilibration time of inhaled anaesthetics. In the model presented here, shunts that reduce fractional pulmonary flow, R–L shunt, lead to reciprocal rises in systemic flow. In turtles, it has been argued that systemic flow is largely maintained, while pulmonary flow reduces in a right-to-left shunt such that right-to-left shunts are associated with lower total $\dot{Q}_{tot}$^6,26^. In this condition, anaesthetic uptake is even slower e.g., 0.5 right-to-left shunt with maintained systemic flow (and therefore reduced $\dot{Q}_{tot}$) requires over double the time to equilibrate, in comparison with the no-shunt condition. Therefore, it is the decrease in pulmonary blood flow which is the main determinant of the effect of shunt on t_90_.

### Implications for measurement of minimum anaesthetic concentration (MAC) in reptiles

MAC is often used to compare anaesthetics, and to set recommendations for anaesthesia in different species. As a puzzling finding, MAC appears to change over the duration of anaesthesia in reptiles^20^, and MAC may change with shunting status^6^. Our model predictions can elucidate these previous findings. Thus, early in anaesthesia, equilibration will not have been reached, therefore higher input partial pressures of anaesthetic are required to attain the arterial and CNS partial pressure required to abolish nociceptive responses. Therefore, there is a higher apparent MAC, which then will reduce as equilibration is attained later in the course of the anaesthetic^5^. For MAC to be determined accurately, the time required between stepwise changes in vaporizer setting must be considerably longer for reptiles, than in mammals. Reptilian MAC studies have also proved difficult to administer due to variability in individual MAC (Bertelsen, personal observation). An assumption of the MAC determination is that end-tidal anaesthetic partial pressures reflects systemic arterial blood partial pressures, which is not the case under conditions of large right-to-left shunts. In a R–L shunting animal, the apparent MAC measured was much higher than the MAC in animals where net right-to-left shunting had been eliminated via infusion of atropine^6^ as also shown in mammals undergoing surgical shunt closure^39^, or in reptiles with very low shunt fractions such as pythons^41^. Variability in individual shunting state may therefore explain a portion of the individual variation in MAC. The use of atropine in selected clinical cases to eliminate net R-L shunts may therefore reduce induction and equilibration times and render anaesthetics more predictable.

### Clinical veterinary implications

Our model predicts that clinical means of supporting $\dot{V}_{L}$ (e.g., artificial ventilation) will hasten equilibration of inhalation anaesthesia. Managing temperature, minimizing surgical blood volume loss, and providing fluid support will prevent unnecessary reductions in $\dot{Q}_{tot}$, and are likely to minimize changes in shunt fraction that could lead to further swings in anaesthetic uptake and elimination rates^42^. In the clinic, while mask or box induction are now increasingly uncommon in domestic mammals, given animal stress and environmental contamination concerns, the uptake of gas anaesthesia in reptiles is hastened by the strategy of inducing at high partial pressures of inhaled agent (e.g., mask or box induction with vaporiser 5% isoflurane, 8% sevoflurane, or higher using open-drop systems^7,43^). This strategy increases the gradient for anaesthetic uptake, and decreases the time required to reach a given absolute concentration (e.g., MAC, or 1.3 MAC)^7,13,44^. Maintaining high vaporizer concentrations and thus high input pressures of anaesthetics is often clinically practiced to buffer against changes in depth of anaesthesia in reptiles caused by e.g., changing intravascular shunts. However, if equilibrated in blood, CNS and heart, these high partial pressures of anaesthetic at induction or maintenance will usually also themselves lead to a reduction in cardiac output. Any cardiovascular depression, i.e. reductions in $\dot{Q}_{tot}$ and reduced tissue perfusion, caused by higher volatile anaesthetic doses will change equilibration times, and thus the immediacy of CNS experience of changes in vaporizer level. Cardiovascular dynamics can also be modified by additional drugs over the period of an anaesthetic: atropine administration is both expected to markedly reduce right-to-left shunts^6^ and usually to prevent vagal reductions in heart rate. Other potential support for $\dot{Q}_{tot}$ and potentially reducing right-to-left shunts could include the use of a β-agonist, such as dobutamine, via increasing cardiac contractility and rate. This will have contributed to the finding that adrenaline administration hastened recovery from inhaled anaesthesia in alligators and chelonians^45–47^. However, large doses of adrenaline may also compromise perfusion and $\dot{Q}_{tot}$ due to the increase in vascular resistance and cardiac afterload given adrenaline’s α effects^32^.

Using multimodal anaesthesia reduces the dependence on the volatile agent alone; animals will be less subject to the cardio-depressive and equilibration lengthening effect of high volatile doses. This is an additional argument for the use of multimodal anaesthesia in practice.

## Summary

In conclusion, the presented model allows nuanced predictions of anaesthetic behavior in a wide range of species, temperatures, anaesthetics and cardiorespiratory variables, useful both in research settings and in predicting clinically relevant anaesthetic outcomes.

## Methods: description of the model

A dynamic multi-compartment model (including pools representing lung, arterial blood, tissue and venous blood: Fig. 1) was created based on^48,49^ assuming:

1. all compartments are represented as uniform well-stirred compartments
2. constant gas capacitance coefficient within each simulation
3. no diffusion limitation at the tissue or lungs
4. no spatial or temporal ventilation-perfusion mismatch in the lungs
5. no heterogeneity in tissue perfusion
6. gas within the lung is fully saturated with water
7. elimination of the anaesthetic is accomplished via the lung

Table 1 details tissue pool volumes, composed from rabbit and terrapin data^19,50^, while blood and tissue anaesthetic partition coefficients were taken from the literature^21,23,28,29,51^. From these, tissue and gas capacitance coefficients (mL_STP_ mL^−1^ mmHg^−1^) were calculated using the gas capacitance coefficient at 37 °C (51.67 µmol L^−1^ mmHg^−1^), except where explicit in the text. The model is based on the equations for each compartment, as detailed in the "Appendix" (Supplemetary infomation). The system of equations were solved numerically in R^52^, version 3.5.1., using the package deSolve^53^ and results visualized in ggplot2^54^.

## Supplementary information

Supplementary Appendix.

## Abbreviations

L: Lung
CL: Lung capillaries
PV: Pulmonary venous blood
LV: Left ventricle/side of heart
SA: Systemic arterial blood
T: Tissues
CT: Tissues capillaries
SV: Systemic venous blood
RV: Right ventricle/side of heart
PA: Pulmonary arterial blood

## List of symbols

$\dot{V}_{L}$: Effective/alveolar lung ventilation
$\beta_{g}$: Air gas capacitance coefficient
$\beta_{b}$: Blood gas capacitance coefficient
$\beta_{T}$: Tissues gas capacitance coefficient
$\dot{Q}_{tot}$: Total cardiac output
$\dot{Q}_{pul}$: Pulmonary blood flow
$\dot{Q}_{sys}$: Systemic blood flow
$\dot{Q}_{R - L}$: Right-to-left shunt blood flow
$\dot{Q}_{L - R}$: Left-to-right shunt blood flow
