# Supplementary material for: Ectothermy and cardiac shunts profoundly slow the equilibration of inhaled anaesthetics in a multi-compartment model
Source: Sci Rep. 2020 Oct 13;10:17157. doi: 10.1038/s41598-020-74014-y (PMC7555730; doi:10.1038/s41598-020-74014-y)
Supplement: Supplementary file 1 — Supplementary Appendix. [file 41598_2020_74014_MOESM1_ESM.pdf]

Ectothermy and cardiac shunts profoundly slow the equilibration of inhaled anaesthetics in a multi-compartment model.

Catherine JA Williams* [a,b,c], Christian Lind Malte [a], Hans Malte [a], Mads F Bertelsen [b] and Tobias Wang[a,d]

[a]Section of Zoophysiology, Department of Biology, Aarhus University, 8000 Aarhus C, Denmark
[b]Center for Zoo and Wild Animal Health, Copenhagen Zoo, Roskildevej 38, 2000 Frederiksberg, Denmark
[c]Ontario Veterinary College, University of Guelph, 50 Stone Road E., Guelph, ON, Canada, N1G 2W1
[d]Aarhus Institute of Advanced Sciences, Aarhus University, 8000 Aarhus C, Denmark

Appendix 1

The model consists of eight coupled differential equation describing how the partial pressures of gas change with time in each of eight interconnected compartments. For a full description of the model and the derivation of the equations see [48,49]. The differential equations for each compartment are given below. Here *P* denotes the partial pressure of gas in a compartment, *V* the volume of that compartment and β the capacitance coefficient for g: gas, b: blood and t: tissue.

Lung + Lung capillaries:
$$\frac{dP_{CL}}{dt} = \frac{\dot{V}_L \beta_g}{V_L \beta_g + V_{CL} \beta_b}(P_L - P_{CL}) + \frac{\dot{Q}_{pul} \beta_b}{V_L \beta_g + V_{CL} \beta_b}(P_{PA} - P_{CL})$$

Pulmonary venous blood
$$\frac{dP_{PV}}{dt} = \frac{\dot{Q}_{pul}}{V_{PV}}(P_{CL} - P_{PV})$$

Left ventricle/side of heart:
$$\frac{dP_{LV}}{dt} = \frac{\dot{Q}_{pul}}{V_{LV}} P_{PV} + \frac{\dot{Q}_{R-L}}{V_{LV}} P_{RV} - \frac{(\dot{Q}_{sys} + \dot{Q}_{L-R})}{V_{LV}} P_{LV}$$

Systemic arterial blood:
$$\frac{dP_{SA}}{dt} = \frac{\dot{Q}_{sys}}{V_{SA}}(P_{LV} - P_{SA})$$

Tissue +tissue capillaries:
$$\frac{dP_{CT}}{dt} = \frac{\dot{Q}_{sys} \beta_b}{V_T \beta_T + V_{CT} \beta_b}(P_{SA} - P_{CT})$$

Systemic venous blood:
$$\frac{dP_{SV}}{dt} = \frac{\dot{Q}_{sys}}{V_{SV}}(P_{CT} - P_{SV})$$

Right ventricle/side of heart:
$$\frac{dP_{RV}}{dt} = \frac{\dot{Q}_{sys}}{V_{RV}} P_{SV} + \frac{\dot{Q}_{L-R}}{V_{RV}} P_{LV} - \frac{(\dot{Q}_{pul} + \dot{Q}_{R-L})}{V_{RV}} P_{RV}$$

Pulmonary arterial blood:
$$\frac{dP_{PA}}{dt} = \frac{\dot{Q}_{pul}}{V_{PA}}(P_{RV} - P_{PA})$$
